# Prevalence and risk factors for CTX-M gram-negative bacteria in hospitalized patients at a tertiary care hospital in Kilimanjaro, Tanzania

**DOI:** 10.1007/s10096-018-3196-8

**Published:** 2018-02-20

**Authors:** Tolbert Sonda, Happiness Kumburu, Marco van Zwetselaar, Michael Alifrangis, Blandina T. Mmbaga, Ole Lund, Frank M. Aarestrup, Gibson Kibiki

**Affiliations:** 1 Kilimanjaro Christian Medical Centre, Kilimanjaro Clinical Research Institute, Moshi, Tanzania; 2 Kilimanjaro Christian Medical University College, Moshi, Tanzania; 3 Centre for Medical Parasitology, Department of Immunology and Microbiology, University of Copenhagen, Copenhagen, Denmark; 4 Department of Infectious Diseases, Copenhagen University Hospital, Copenhagen, Denmark; 5 Centre for Biological Sequence Analysis, Technical University of Denmark, Lyngby, Denmark; 6 DTU-Food, Centre for Genomic Epidemiology, Technical University of Denmark, Lyngby, Denmark; 7 East African Health Research Commission, Bujumbura, Burundi

**Keywords:** ESBL, Gram-negative bacteria, Prevalence, CTX-M, Whole genome sequencing, Tanzania

## Abstract

Emergence and spread of extended spectrum beta-lactamase (ESBL)-producing gram-negative bacteria, mainly due to CTX-M, is a major global public health problem. Patients infected with ESBL-producing gram-negative bacteria have an increased risk of treatment failure and death. We investigated the prevalence and risk factors for CTX-M gram-negative bacteria isolated from clinical specimens of patients hospitalized at a tertiary care hospital in Kilimanjaro, Tanzania. Isolated gram-negative bacteria from inpatients admitted at Kilimanjaro Christian Medical Centre (KCMC) between August 2013 and August 2015 were fully genome sequenced. The prevalence of ESBL-producing gram-negative bacteria was determined based on the presence of *bla*_CTX-M_. The odds ratio (OR) and risk factors for ESBL-producing gram-negative bacteria due to CTX-M were assessed using logistic regression models. The overall CTX-M prevalence (95% CI) was 13.6% (10.1–18.1). Adjusted for other factors, the OR of CTX-M gram-negative bacteria for patients previously hospitalized was 0.26 (0.08–0.88), *p* = 0.031; the OR for patients currently on antibiotics was 4.02 (1.29–12.58), *p* = 0.017; the OR for patients currently on ceftriaxone was 0.14 (0.04–0.46), *p* = 0.001; and the OR for patients with wound infections was 0.24 (0.09–0.61), *p* = 0.003. The prevalence of ESBL-producing gram-negative bacteria due to CTX-M in this setting is relatively low compared to other previous reports in similar settings. However, to properly stop further spread in the hospital, we recommend setting up a hospital surveillance system that takes full advantage of the available next-generation sequencing facility to routinely screen for all types of bacterial resistance genes.

## Introduction

Existence of antibiotic resistance due to extended spectrum beta-lactamase (ESBL)-producing gram-negative bacteria is a major global public health problem [1] and has been reported in all regions of the world [2–7]. Compared to patients infected with beta-lactam susceptible bacteria, patients infected with ESBL-producing gram-negative bacteria put a greater burden on health-care resources and have an increased risk of treatment failure and poor outcomes including death [8, 9]. High-income countries (HICs) have surveillance systems in place to estimate the burden of bacterial infections due to ESBL-producing gram-negative bacteria, and to determine risk factors for acquisition of ESBL-producing gram-negative bacteria as well as the clinical outcomes associated with infection [1, 10–12]. In Sub-Sahara Africa (SSA), data on ESBL-producing gram-negative bacteria epidemiology and risk factors associated with ESBL-producing gram-negative bacteria infection are scarce. When available, the data mostly originates from hospital-based studies [13–18]. Several risk factors have been documented to be associated with ESBL-producing gram-negative bacteria acquisition, including previous hospitalization, previous use of antibiotics such as third-generation cephalosporins, hospital overcrowding, bed sharing when hospitalized, and international travel [19–25].

In 2002, Blomberg et al. reported high proportions of infections due to ESBL-producing gram-negative bacteria at the National Hospital in Dar es Salaam, Tanzania [26]*.* Since then, other reports have shown an increasing trend of ESBL-producing gram-negative bacteria infections in the same hospital [14, 27–29]. Similarly, studies conducted at a tertiary hospital in Mwanza, Tanzania have documented an increase in ESBL-producing gram-negative bacteria infections [28, 30, 31]. Like the centers in Dar es Salaam and Mwanza, Kilimanjaro Christian Medical Centre (KCMC) is a major zonal tertiary healthcare facility, serving nearly one quarter of Tanzania’s estimated 53.5 million inhabitants [32]. Its setting however sets KCMC apart: the Kilimanjaro region in Tanzania has among the highest international movements of people in the country for tourism and business. The region has four distinct ecosystems inhabited by diverse communities of miners, pastoralists, agrarians, and fishermen. Given the public health importance of ESBL-producing gram-negative bacteria, the role that KCMC plays in guiding empirical treatment, and the need for therapeutic decisions to be based on guidelines which are derived from local epidemiological data [33], a study on ESBL-producing gram-negative bacteria in KCMC was found to be imperative. In our setting, little attention is paid to gram-negative bacteria, although they have been reported in east Africa and elsewhere to be causes of outbreaks and to be main contributors of ESBL resistance due to production of CTX-M [13, 34]. The aim of this study was to determine the proportion estimates and risk factors for ESBL-producing gram-negative bacteria due to CTX-M using whole genome sequences of all *gram-negative bacteria* isolated from clinical specimens at KCMC hospital.

## Materials and methods

### Study design, participants, and specimen collection

A hospital-based cross-sectional study was conducted at KCMC between August 2013 and August 2015. Geographically, KCMC is located in north-eastern Tanzania and serves as a zonal referral hospital. The hospital has a bed capacity of 650 with approximately 500 outpatients seeking medical services daily.

The study involved patients admitted in medical, surgical, and pediatrics wards who were suspected to have bacterial infection. A written consent was obtained from each participant or from parents or guardians of children before enrolment into the study. Clinical specimens collected for bacterial culture were sputum, wound or pus swab, stool, and blood.

### Bacterial culture and identification

Bacteria culture, isolation, and identification were performed according to in-house standard operating procedures as well as the Clinical and Laboratory Standards Institute (CLSI) guidelines as described by Kumburu et al. [35].

### Genomic DNA isolation, whole genome sequencing, and analysis

Bacterial genomic DNA (gDNA) was purified and its concentration determined using the Easy-DNA extraction kit Invitrogen® and the Qubit dsDNA Assay Kit Invitrogen® respectively. The gDNA library preparation was performed following Nextera® XT DNA Sample Preparation Guide [36]. In brief, each gDNA was tagmented (tagged and fragmented) by the Nextera® XT transposome. The transposome simultaneously fragments the input DNA and adds adapter sequences to the ends. Thereafter followed a limited-cycle PCR amplification whereby indexes required for cluster formation were added to each DNA piece. Then each gDNA library was normalized to ensure equal representation during sequencing. Equal volumes of the normalized library were combined, diluted in hybridization buffer, and heat-denatured prior to sequencing on the Illumina MiSeq platform (Illumina, Inc., San Diego, CA). Whole genome sequence reads were analyzed using bioinformatics tools available on https://cge.cbs.dtu.dk/services/. For this report’s purpose, the analyses included de novo reads assembly, species identification, and determination of *bla*_CTX-M_ encoding ESBL.

### Collection of metadata and statistical analyses

Case report and laboratory result forms designed for this study were used to collect metadata. Double data entry was performed in OpenClinica (OpenClinica LLC, MA USA). Stata version 13 (StataCorp LP, Texas 77845 USA) was used for all statistical analyses. The prevalence of resistance due to ESBL-producing gram-negative bacteria was calculated as the number of gram-negative bacteria positive for *bla*_CTX-M_ divided by total number of gram-negative bacteria sequenced. The prevalence across levels of a categorical variable, such as age, gender, wards admitted, type of bed, hospitalization, antibiotics use, disease conditions, and comorbidities, was compared using Chi-square test or Fisher’s exact test. Logistic regression was performed to determine risks of isolating CTX-M gram-negative bacteria strains from patients. In the multivariate analysis, we included any variable whose *p* value ≤ 10% and those that were considered key risk factors regardless of their *p* values being > 10% in univariate analysis. Statistical significance of associations was decided based on a two-tailed *p* value and respective 95% confidence intervals.

## Results

### Study population

In total, 575 patients were included in this study (Table 1). These baseline data have also been presented before by Kumburu et al. [35]. The median age in years (IQR) was 43 (30–57), and 348 (60.6%) of the patients were males. Three hundred thirty nine (59.2%) had primary education level and 271 (47.3%) were farmers. A total of 301 (52.3%) patients were admitted into medical wards. One hundred six (18.4%) of all patients were on stretcher type beds. A total of 287 (49.9%) specimens were wound and/or pus swabs.

**Table 1:** Demographic and clinical characteristics of the study population

Patient characteristic	Total
Number of patients	575 (100)
Median age in years (IQR)	43 (30–57)
Gender	
Female	226 (39.4)
Male	348 (60.6)
Occupation	
Farming	271 (47.3)
Employed	177 (30.9)
Other	125 (21.8)
Education level	
No	130 (22.7)
Primary	339 (59.2)
Secondary/Above	104 (18.2)
Ward of admission	
Surgical	257 (44.7)
Medical	301 (52.3)
Other	17 (3.0)
Location of patient	
In Corridor/Stretcher	106 (18.4)
Inside room/Bed	469 (81.6)
Specimen collected	
Blood	117 (20.3)
Sputum	120 (20.9)
Stool	51 (8.9)
Swabs (wound or pus)	287 (49.9)
Comorbid conditions	
Wound	263 (45.9)
Pneumonia	81 (14.1)
Tuberculosis	60 (10.5)
Sepsis, septicemia	113 (19.7)
Diarrhea	20 (3.5)
Diabetes	122 (21.3)
HIV	81 (14.1)
Cancer	52 (9.1)
Currently on antibiotics	412 (71.9)

A total of 263 (45.9%) patients had infected wounds, 81 (14.1%) had pneumonia, and 20 (3.5%) had diarrhea. A total of 60 (10.5%) were TB, 81(14.1%) were HIV, and 122 (21.3%) were diabetic cases. Out of all participants, 412 (71.9%) were on antibiotics at the time specimens were collected (Table 1).

### Prevalence of CTX-M in gram-negative bacteria

A total of 590 specimens were collected in the following distribution**:** 56 were stool, 122 were sputum, 126 were blood, and 286 were wound or pus swabs. Of all the specimens, 249 were culture-positive yielding 377 bacterial isolates that were fully sequenced. Of these, 287 bacterial isolates were gram-negative bacteria. The most prevalent gram-negative bacteria were *Proteus* spp. (*n* = 48, 16.7%), *Escherichia coli* (*n* = 44, 15.3%), *Pseudomonas* spp. (*n* = 40, 13.9%), and *Klebsiella* spp. (*n* = 38, 13.2%). The CTX-M gene was found in 39 of the 287 gram-negative bacteria, yielding an overall prevalence (95% CI) of 13.6% (10.1–18.1) (Table 2). The prevalence in isolates from those admitted in surgical, medical, and ICU wards, respectively, were 5.0% (2.5–9.8), 9.4% (4.7–17.8), and 3.1% (0.4–19.8). Table 2 shows also crude odds ratios (OR) for different patient characteristics. The crude OR (95% CI) of CTX-M gram-negative bacteria in patients admitted in medical wards was significantly higher compared to those from surgical wards; OR 3.62 (1.74–7.52), *p* = 0.001. Bacterial isolates from those sleeping in ward corridors/stretchers had a prevalence of 7.4% (3.9–13.7), versus 4.8% (2.4–9.4) for those sleeping inside rooms/beds (Table 2). Prevalence of ESBL-producing gram-negative bacteria due to CTX-M per different disease conditions was investigated (Table 3). Among patients with wound infections, the prevalence was 4.2% (2.0–8.6) while among diarrhea cases, TB cases, and patients with sepsis, the prevalence was 2.0% (4.6–56.3), 6.7% (0.9–37.1), and 2.9% (0.7–11.3), respectively. Furthermore, ESBL-producing gram-negative bacteria prevalence due to CTX-M in HIV and in diabetic cases was 11.1% (3.5–30.0) and 8.5% (3.8–17.7), respectively.

**Table 2:** Socio-demographic characteristics of patients from which CTX-M gram-negative bacteria were isolated

Patient characteristics	CTX-M gram-negative bacteria		
	Prevalence (95% CI)	Crude OR (95% CI)	*P* value
All CTX-M	13.6 (10.1–18.1)	–	–
Age (years)			
0–35(*n* = 87)	4.6 (1.7–11.7)	1 (Ref)	–
36–53(*n* = 84)	6.0 (2.5–13.6)	1.27 (0.53–3.01)	0.595
54 and above (*n* = 100)	8.0 (4.0–15.3)	1.12 (0.48–2.63)	0.786
Department^a^			
SW (*n* = 159)	5.0 (2.5–9.8)	1 (Ref)	–
MW (*n* = 85)	9.4 (4.7–17.8)	3.62 (1.74–7.52)	0.001
ICU (*n* = 32)	3.1 (0.4–19.8)	0.69 (0.15–3.20)	0.636
Hospital stay (days)			
0–4 (*n* = 82)	7.3 (3.3–15.5)	1 (Ref)	–
5–12 (*n* = 91)	6.6 (3.0–14.0)	1.05 (0.47–2.36)	0.911
13 and above (*n* = 86)	3.5 (1.1–10.4)	0.47 (0.18–1.25)	0.129
Gender			
Female (*n* = 111)	4.5 (1.9–10.4)	1 (Ref)	–
Male (*n* = 166)	7.2 (4.1–12.3)	0.91 (0.45–1.82)	0.783
History of hospitalization			
No (*n* = 188)	6.4 (3.6–10.9)	1 (Ref)	–
Yes (*n* = 99)	5.1 (2.1–11.7)	0.53 (0.24–1.16)	0.111
History of medication			
No (*n* = 109)	3.7 (1.4–9.4)	1 (Ref)	–
Yes (*n* = 178)	7.3 (4.3–12.2)	1.26 (0.62–2.58)	0.521
Current medication			
No (*n* = 81)	8.6 (4.1–17.2)	1 (Ref)	–
Yes (*n* = 206)	4.9 (2.6–8.8)	1.0 (0.47–2.12)	0.998
Patient location			
Inside room/Beds (n = 166)	4.8 (2.4–9.4)	1 (Ref)	–
Corridor/Stretcher (*n* = 121)	7.4 (3.9–13.7)	0.94 (0.48–1.88)	0.877

^a^*sw* surgical ward, *mw* medical ward, *ICU* intensive care unit

**Table 3:** Morbidity types and prevalence of CTX-M gram-negative bacteria

Patient characteristics	CTX-M gram-negative bacteria		
	Prevalence (95% CI)	Crude OR (95% CI)	*P* value
Infected wounds			
No (*n* = 109)	9.2 (4.9–16.3)	1 (Ref)	–
Yes (*n* = 167)	4.2 (2.0–8.6)	0.28 (0.14–0.58)	0.001
Pneumonia			
No (*n* = 261)	6.1 (3.8–9.8)	1 (Ref)	–
Yes (*n* = 15)	6.7 (0.9–37.1)	1.61 (0.43–6.01)	0.475
Tuberculosis			
No (*n* = 261)	6.1 (3.8–9.8)	1 (Ref)	–
Yes (*n* = 15)	6.7 (0.9–37.1)	0.96 (0.21–4.44)	0.96
Sepsis			
No (*n* = 220)	6.8 (4.1–11.0)	1 (Ref)	–
Yes (*n* = 67)	2.9 (0.7–11.3)	0.56 (0.22–1.39)	0.211
Diarrhea			
No (*n* = 266)	5.6 (3.4–9.2)	1 (Ref)	–
Yes (*n* = 10)	2.0 (4.6–56.3)	10.97 (2.94–40.98)	0.001
Diabetes			
No (*n* = 205)	5.4 (2.9–9.5)	1 (Ref)	–
Yes (*n* = 71)	8.5 (3.8–17.7)	1.04 (0.48–2.26)	0.928
HIV			
No (*n* = 249)	5.6 (3.3–9.3)	1 (Ref)	–
Yes (*n* = 27)	11.1 (3.5–30.0)	2.46 (0.96–6.30)	0.06

Table 3 further documents crude ORs for different morbidities. Crude OR of CTX-M gram-negative bacteria in patients with wounds was significantly lower compared to those who did not have wounds; OR 0.28 (0.14–0.58), *p* = 0.001, while the crude OR of CTX-M gram-negative bacteria in patients with diarrhea was significantly higher compared to those without; OR 11.0 (2.94–40.98), *p* = 0.001. The trend for OR of infection with CTX-M gram-negative bacteria among HIV cases was higher compared to non-HIV cases; OR 2.46 (0.96–6.30), *p* = 0.06.

We also investigated the prevalence of CTX-M gram-negative bacteria in patients on different antibiotics (Table 4). The prevalence of CTX-M gram-negative bacteria in patients on at least one antibiotic was 6.8% (4.1–10.9). The OR of CTX-M gram-negative bacteria when patients were on other Ceftriaxone as compared to those who were not showed that those on Ceftriaxone had a reduced risk; OR 0.34 (0.16–0.71), *p* = 0.004, while there was a trend that those on Ciprofloxacin had an increased risk of CTX-M gram-negative bacteria; OR 2.62 (0.88–7.83), *p* = 0.084.

**Table 4:** Antibiotic use and prevalence of CTX-M in gram-negative bacteria

Patient characteristics	CTX-M gram-negative bacteria		
	Prevalence (95% CI)	Crude OR (95% CI)	*P* value
Currently on antibiotics			
No (*n* = 54)	3.7 (0.9–13.9)	1 (Ref)	–
Yes (*n* = 222)	6.8 (4.1–10.9)	1.35 (0.53–3.41)	0.529
Ciprofloxacin			
No (*n* = 258)	6.2 (3.8–9.9)	1 (Ref)	–
Yes (*n* = 18)	5.6 (0.7–32.1)	2.62 (0.88–7.83)	0.084
Ceftriaxone			
No (*n* = 135)	8.1 (4.5–14.2)	1 (Ref)	–
Yes (*n* = 141)	4.3 (1.9–9.2)	0.34 (0.16–0.71)	0.004
Cotrimoxazole			
No (*n* = 267)	6.0 (3.7–9.6)	1 (Ref)	–
Yes (*n* = 9)	11.1 (1.3–53.4)	3.31 (0.79–13.86)	0.101
Cloxacillin			
No (*n* = 216)	6.0 (3.5–10.1)	1 (Ref)	–
Yes (*n* = 60)	6.6 (2.5–16.6)	0.64 (0.25–1.61)	0.342
Metronidazole			
No (*n* = 256)	5.9 (3.2–10.7)	1 (Ref)	–
Yes (*n* = 20)	6.5 (3.1–13.1)	1.63 (0.52–5.17)	0.405

### Risk factors for CTX-M gram-negative bacteria

Table 5 shows adjusted OR (AOR) for various patient characteristics. After adjusting for other patient characteristics, several variables appeared to be statistically associated with CTX-M gram-negative bacteria. These variables include prior admission, currently on antibiotics, currently on ceftriaxone, and wound infections. Those with a prior admission history showed a relatively reduced risk of CTX-M gram-negative bacteria compared to those with no prior history of admission; AOR 0.26 (0.08–0.88), *p* = 0.031. An increased risk of CTX-M gram-negative bacteria was observed for those currently on at least one antibiotic compared to those who were not on any antibiotics; AOR 4.02 (1.29–12.58), *p* = 0.017. A decreased risk of CTX-M gram-negative bacteria was observed among those who were currently on Ceftriaxone compared to those who were not on Ceftriaxone; AOR 0.14(0.04–0.46), *p* = 0.001 and for those with wound infections was 0.24 (0.09–0.61), *p* = 0.003. There is also seen a lower likelihood in patients with wound infection than those without wound infections.

**Table 5:** Multivariate logistic regression model showing adjusted ORs for isolating CTX-M gram-negative bacteria

Patient characteristics	AOR^a^ (95% CI)	*P* value
Prior admission	0.26 (0.08–0.88)	0.031
Prior medication	1.50 (0.62–3.62)	0.368
Currently admitted in surgical ward	0.70 (0.28–1.76)	0.444
Patient inside the room	0.76 (0.32–1.78)	0.522
Over 4 days of hospitalization	0.72 (0.31–1.71)	0.462
Currently on antibiotic	4.02 (1.29–12.58)	0.017
Currently on Ciprofloxacin	0.84 (0.21–3.35)	0.804
Currently on Ceftriaxone	0.14 (0.04–0.46)	0.001
HIV positive	1.15 (0.37–3.55)	0.804
Wound infection	0.24 (0.09–0.61)	0.003

^a^Adjusted odds ratio

## Discussion

This survey investigated the prevalence and possible risk factors for CTX-M gram-negative bacteria in hospitalized patients in Kilimanjaro, Tanzania. It is the first report to investigate ESBL-producing gram-negative bacteria due to CTX-M in clinical specimens using whole genome sequencing and not based on phenotypic testing. To the best of our knowledge, it is the first risk factor analysis of ESBL-producing gram-negative bacteria infections in Tanzania. The overall prevalence of CTX-M gram-negative bacteria in this study was 13.6%. This hospital-study’s findings are comparable with other hospital studies that had previously reported low ESBL-producing gram-negative bacteria prevalence in Gabon, 15.0% [37], and Central Africa Republic, 4 to 19% [38]. However, the report’s prevalence contrasts with 42% prevalence in a review on general ESBL-producing gram-negative bacteria in East Africa hospitals [34], a 29.7% prevalence previously reported in Kilimanjaro by Kajeguka et al. [39], and 32.6% prevalence reported in Guinea-Bissau [22]. Further, our findings contrast with other reports on ESBL-producing gram-negative bacteria in hospitals outside the East African region in that they had shown higher prevalence than in this report: Cameroon 55% [19], Ghana 49% [23], Egypt above 45% [40], and South Africa 83% [41]. There could be several reasons for the observed low prevalence (13.6%) of CTX-M gram-negative bacteria, including the fact that the majority of patients were sampled while they had been on antibiotics for a number of days since admission. The prevalence of CTX-M gram-negative bacteria was observed to decrease with hospitalization days. The use of ceftriaxone in the majority of inpatients at KCMC is a plausible explanation for the observed low prevalence of CTX-M, and the decrease in prevalence over time. Moreover, the prevalence of CTX-M at admission may be relatively high due to selection of ESBL-producing gram-negative bacteria in the community due to overuse of antibiotics other than ceftriaxone. A comparison of CTX-M resistance between patients on ceftriaxone versus patients on other cephalosporins like ceftazidime or cefepime would have been of great interest; however, the available data was insufficient for this analysis.

Prevalence of CTX-M gram-negative bacteria appeared to increase with age. The numbers of patients with age more than 65 years in this study were low, so the comparison with other publications could not be done. Other reports [42–44] have shown adults over 65 to be almost three times at risk of infection with ESBL-producing gram-negative bacteria. Due to their diminishing immunity and repeated hospitalization, this group is more prone to infections including resistant bacterial strains. However, there appears to be no statistical evidence to support the observed proportion differences in the risks of the CTX-M gram-negative bacteria across age groups.

We investigated whether or not sleeping on stretchers and sleeping in corridors as proxy indicators of overcrowding were associated with the CTX-M gram-negative bacteria. It was found that the prevalence of CTX-M gram-negative bacteria in patients sleeping in ward corridors (on stretchers) as the proper beds were all occupied and no space inside the rooms were relatively high compared to their counterparts. However, sleeping on stretchers and sleeping in corridors were not significantly associated with increased risk of isolating CTX-M gram-negative bacteria.

Factors that showed statistically significant associations with CTX-M gram-negative bacteria include prior admission, currently on antibiotics, use of Ceftriaxone, and wound infections. This report shows that a large proportion (71.9%) of patients was already on antibiotics therapy when specimens were collected for microbiological analyses. Prevalence of isolating CTX-M gram-negative bacteria from patients who were on at least one type of antibiotics was higher (6.8%) than in those who were not (3.7%). The likelihood of isolating CTX-M gram-negative bacteria from those who were currently on antibiotics was found to be four times higher than from those who were not on antibiotics therapy. Reports have shown that the risk of developing antibiotics resistance in societies where antibiotics are excessively used is high [44, 45] due to antimicrobial resistance selection pressure. In KCMC, being a referral hospital, the patients coming to seek medical services are those who might have used antibiotics but treatment failed due to carriage of resistant strains prior to their referrals. Furthermore, given the fact that patients are referred to our hospital from different healthcare facilities, the likelihood of patients to have been on antibiotics is high. Self-referred patients rarely have a paper record of prior medications. Empirical therapy, self-medication, and over-the-counter medications are common in low-income countries (LIC) [46–48]. This experience-based therapy practiced by physicians in LIC is one of the factors fuelling emergence of resistance, particularly when unnecessarily switching to higher generations of antibiotics.

Ceftriaxone has been a drug of choice by many physicians in our setting due to its broad-spectrum activity. Over 51% of the analyzed gram-negative bacteria were isolated from patients who were on Ceftriaxone. The prevalence of CTX-M gram-negative bacteria was 4.3% from patients who were on Ceftriaxone, compared to 8.1% for those who were not. The likelihood of isolating CTX-M gram-negative bacteria from those who were currently on Ceftriaxone appeared to be seven times lower than those who were not treated with Ceftriaxone. As self-medication with Ceftriaxone is uncommon due to its high cost, this suggests that treatment with injectable Ceftriaxone in the hospital was effective against CTX-M gram-negative bacteria*.* Nevertheless, its prescription should be guided by microbiological results.

A substantial proportion (34.5%) of CTX-M gram-negative bacteria was isolated from patients who had a history of admission (hospitalization). The prevalence of isolating CTX-M gram-negative bacteria from patients with no history of admission (hospitalization) was 6.4%, higher than 5.1% in those with hospitalization history. However, the likelihood of isolating CTX-M gram-negative bacteria from patients with history of admission was approximately four times lower than their counterparts, consistent with a recent report [49] that showed a 32% lower risk of ESBL-producing gram-negative bacteria among previously hospitalized patients. It could be that during past admissions, these patients were put on Ceftriaxone doses that had successfully treated CTX-M gram-negative bacteria. This report suggests that Ceftriaxone could be effective against CTX-M gram-negative bacteria*.* Furthermore, the observed high risk of CTX-M gram-negative bacteria among those who had no prior admission could be explained by the fact that they were carrying resistant CTX-M gram-negative bacteria from the community. However, these findings are different from reports [20, 45, 50, 51] which found that previous admission and use of cephalosporins increase the risk of CTX-M gram-negative bacteria*.*

A benefit of this study is its contribution to a larger project in which we have been able to build an infrastructure and implement next-generation sequencing (NGS) techniques for clinical diagnostics. For the current report, we have been able to analyze clinical specimens by doing whole genome sequencing to identify CTX-M genes that code for ESBL. We acknowledge that several limitations need to be addressed. Firstly, wound and pus swab specimens make up the majority of samples studied. Microbiology results do not always represent the cause of the infection. Secondly, in this report, ESBL-producing gram-negative bacteria resistance was defined based on the presence of the CTX-M gene. Despite its important role in resistance, this restricted definition means we may have underestimated the ESBL-producing gram-negative bacteria prevalence. Underestimation of ESBL-producing gram-negative bacteria resistance could also be attributed to sampling patients who were already on antibiotics treatment. Thirdly, though our report highlights some key aspects regarding prevalence and risk factors for ESBL-producing gram-negative bacteria resistance due to CTX-M gram-negative bacteria that are important in programming for antimicrobial control, the study was insufficiently powered to confidently draw conclusions about all suspected variables, especially those that appeared contradicting. Fourth, this study was unable to differentiate between community-acquired and hospital-acquired ESBL-producing gram-negative bacteria as clinical sampling was done during, rather than prior to hospitalization. Finally, the widespread use of antibiotics in our settings makes it difficult to ascertain whether or not exposure to antibiotics preceded the emergency of ESBL-producing gram-negative bacteria resistance due to CTX-M.

## Conclusions

In conclusion, although the prevalence of ESBL-producing gram-negative bacteria due to CTX-M in this setting was found to be low, it is worthwhile to devise approaches aimed at containing the situation before it gets out of control. To properly stop their spread in the hospital, apart from direct containment methods, we recommend setting up a hospital surveillance system that takes full advantage of the available NGS facility to routinely screen for other resistant bacterial genes.
